# The influence of different surface texture parameters on the cell proliferation and adhesion early behaviors on the surface of titanium alloy

**DOI:** 10.3389/fbioe.2026.1679515

**Published:** 2026-02-03

**Authors:** Fuqiang Lai, Changsheng Cao, Linfeng Xie, Mingxuan Shang, Wenge Liu, Yumei Li, Zhihuang Qiu, Liangwan Chen

**Affiliations:** 1 Department of Cardiovascular Surgery, Fujian Medical University Union Hospital, Fuzhou, Fujian, China; 2 Key Laboratory of Cardio-Thoracic Surgery (Fujian Medical University), Fujian Province University, Fuzhou, Fujian, China; 3 School of Mechanical Engineering and Automation, Fuzhou University, Fuzhou, Fujian, China; 4 Department of Orthopedics, Fujian Medical University Union Hospital, Fuzhou, Fujian, China; 5 Fujian Center for Safety Evaluation of New Drug, Fujian Medical University, Fuzhou, Fujian, China

**Keywords:** cell proliferation and adhesion, femtosecond laser processing, parameter optimization, surface texture, titanium alloy

## Abstract

Current clinical practice has extensively validated the efficacy of left ventricular assist devices (LVADs) in managing end-stage heart failure. A persistent challenge across all ventricular assist systems involves achieving optimal biocompatibility at the critical interface between the LVAD outlet and myocardial tissue. In this study, femtosecond laser processing (FLP) technology was utilized to engineer microtextured surfaces with controlled geometric parameters on the titanium alloy surface. The experimental design systematically assessed surface morphology and compositional variations for four distinct patterns (circular, triangular, square, hexagonal) and three texture depths (10, 20, 40 μm). FLP demonstrated favourable microstructural fabrication quality, producing defined pattern boundaries with minimal thermal impact on adjacent regions. While all textured surfaces exhibited characteristic periodic processing marks at their bases, increased texture depth correlated with progressive roughness amplification in these basal regions. Elemental analysis revealed that oxygen enrichment specifically along texture peripheries compared to untextured surfaces. Cellular early response studies demonstrated that surface texturing significantly enhanced cardiac fibroblasts adhesion on titanium substrates while concurrently modifying fibroblast growth patterns. Quantitative analysis identified 20 μm as the optimal texture depth for cellular proliferation and adhesion, outperforming both shallower (10 μm) and deeper (40 μm) configurations. Geometric comparisons indicated that square patterns induced the best pronounced pro-proliferative effects, followed by hexagonal patterns. Mechanistic observations suggest that surface micro-roughness facilitates initial cell adhesion, with subsequent proliferation biodynamics being governed by topographical guidance effects. These findings establish clear structure-function relationships between engineered surface parameters and biological responses, providing significant insights for LVAD surface treatment and optimization.

## Highlights

- Micro-textures were introduced to the left ventricular assist device inlet surface.
- Micro-textures are processed using femtosecond laser processing technology.
- Biocompatibility evaluation and cell adhesion behavior analysis were conducted.
- When the texture depth of 20 μm, the cell proliferation performance is optimal.
- Square textures show better early proliferation behavior than circle, triangle and hexagon.

## 1 Introduction

A ventricular assist device (VAD) is a type of medical mechanical system used to assist or even replace the function of the heart in pumping blood. As a form of artificial heart component, ventricular assist devices play a significant role in treating end-stage heart failure. Due to the fact that the left ventricle bears higher pressure and is more prone to failure compared to the right ventricle, left ventricular assist devices (LVADs) have been widely utilized (Long et al., 2019; Kannojiya et al., 2021).

Contemporary research on left ventricular assist devices (LVAD) surface treatments identifies has six clinically relevant or developmentally promising approaches: titanium nitride (TiN) coatings (Harman et al., 1997), diamond-like carbon (DLC) films (Cui and Li, 2000), heparin-based coatings, methacryloyloxyethyl phosphorylcholine (MPC) polymer layers (Nakabayashi and Williams, 2003), engineered textured surfaces, and endothelial cell linings (Wang et al., 2025). The suboptimal hemocompatibility of conventional biomaterials has driven, the advancement of surface engineering solutions aimed at mitigating thromboembolic complications and infection risks (Li et al., 2024). Surface coatings applied to LVAD components have evolved from passive organic/inorganic systems to bioactive interfaces incorporating biological molecules or cellular components. TiN coatings, DLC films, and surface texturing represent inorganic modification strategies, while MPC polymers constitute organic coatings. Bioactive approaches include heparin immobilisation techniques (Tan et al., 2003) and EC lining technologies (Fujisawa et al., 2000).

Among the various passive coatings, the DLC coating is more advanced and has better performance than the TiN coating. However, the MPC polymer coating is not suitable for long-term or permanent implantation in VADs, but they usually exhibit better blood compatibility than the inorganic TiN and DLC coatings. An appropriate textured surface has the ability to induce the formation of an EC layer in the body, thereby contributing to good blood compatibility. If the surface has specially designed porosity, roughness or pattern textures, then the surface can attract proteins and ECs, and form a layer *in situ* that is essentially compatible with blood after implantation. Current commercial VADs predominantly utilize the first five coating types, with surface functionalization strategies employing two distinct mechanisms. Passive coatings such as TiN, DLC, and MPC polymers create physical barriers between blood components and substrate materials. In contrast, active coatings, including heparin-modified surfaces, textured interfaces, and EC linings, directly interact with biological systems through anticoagulant activity or modulation of vascular re-modelling processes. Among passive coating technologies, DLC coatings demonstrate superior properties and superior performance compared to conventional TiN coatings. While MPC polymer coatings exhibit enhanced hemocompatibility relative to inorganic alternatives, their limits durability restricts application to temporary VAD implants rather than permanent devices.

Engineered textured surfaces present unique advantages through their capacity to induce spontaneous endothelialization *in vivo*. Surfaces with optimized microtopographical features-including controlled porosity, specific roughness parameters, or patterned geometries-demonstrate selective protein adsorption and EC recruitment capabilities (Petrović et al., 2019). This biological response facilitates the *in-situ* formation of a natural blood-compatible interface post-implantation, effectively creating a self-sustaining hemocompatibility layer through endogenous biological processes (Zhuang et al., 2016). The progressive maturation of such EC monolayers on textured surfaces holds significant potential for addressing chronic complications in long-term LVAD support.

The interfacial interactions between cardiac tissue and device surfaces in LVADs initiate a cascade of complex biological events encompassing cellular adhesion, activation, and proliferation (Gupta et al., 2025). This biomaterial-host interplay begins with the rapid adsorption of extracellular membrane proteins onto device surfaces - the primary biological response during cell-surface interactions. These adsorbed proteins undergo conformational activation and mediate subsequent biological reactions through catalytic regulation or signalling modulation (Tanné et al., 2010).

Biological responses *in vivo* constitute dynamically regulated processes influenced by multiple interfacial parameters, with surface characteristics of biomaterials serving as critical determinants of biological outcome trajectories. Key surface properties, including chemical composition, surface topography, surface free energy, elasticity, and charge distribution, collectively modulate protein-cell interactions that ultimately govern cellular and protein adhesion patterns (Motomura et al., 2019). For instance, platelet adhesion and activation on bio-material surfaces demonstrate direct correlation with surface energy profiles, charge states, and chemical constituents. However, these thrombotic responses are principally mediated through adsorbed proteins such as fibrinogen rather than direct surface effects.

The formation of a plasma-derived “conditioning film” through instantaneous protein adsorption following blood contact creates an interfacial buffer layer. This protein interphase significantly attenuates the direct influence of inherent surface properties on cellular responses. While surface characteristics dictate initial protein adsorption patterns, the subsequent biological cascade becomes progressively governed by the adsorbed protein layer’s biochemical signalling. This dual-phase interaction mechanism-comprising immediate surface-mediated protein recruitment followed by protein-guided cellular responses-establishes critical design parameters for optimizing LVAD hemocompatibility through surface engineering strategies (Zhang et al., 2025).

Current research has extensively investigated the impact of titanium alloy surface texturing on cellular adhesion behavior. Huang et al. (Yang et al., 2021) developed a multi-step surface engineering protocol combining cathodic arc ion plating of TiZrTa films with subsequent laser texturing oxidation to enhance the biocompatibility of Ti-6Al-4V substrates. The laser texturing oxidation process generated structured microtopography with regular groove patterns, including linear, circle, and grid configurations. while simultaneously inducing surface oxidation and mixed oxide formation in the TiZrTa coating. Microstructural analysis revealed substantial surface modification through laser patterning, with distinct morphological transitions between treated and untreated regions.

Antibacterial performance evaluation against *Staphylococcus aureus* demonstrated no significant antibacterial advantage for laser texturing oxidation treated specimens during 12-h or 24-h culture periods. This limited antibacterial efficacy was attributed to the composite nature of laser-textured surfaces comprising both coating and substrate materials. However, cellular compatibility assessments revealed enhanced biological performance. Laser texturing oxidation treated coatings significantly improved viability rates (48-h culture) of mouse fibroblasts L-929, particularly in specimens with linear and circular patterns at 50 μm spacing intervals. Extended evaluation using MG-63 human osteosarcoma cells showed comparable viability levels across all specimens after 2-week culture periods, indicating favorable biocompatibility of TiZrTa-coated Ti-6Al-4V with both cell types.

These findings demonstrate the dual-phase nature of surface-engineered titanium alloys: while antibacterial properties remain challenging to optimize through texturing alone, controlled micro-topographical modification effectively enhances cellular biocompatibility. The study highlights the critical relationship between pattern geometry (particularly spacing dimensions) and biological response modulation, providing valuable insights for designing LVAD surfaces that balance hemocompatibility with tissue integration requirements.

Laser surface texturing of titanium alloys has been widely applied in bone implant technology, with recent investigations focusing on optimizing surface characteristics for enhanced biological performance. Wang et al. (Xu et al., 2018) systematically explored ultraviolet laser processing techniques to create titanium surfaces with varying edge radii, bioinspired egg-trap configurations, and disordered rough microstructures, revealing critical relationships between laser parameters, surface properties, and cellular behavior. During laser processing, elemental migration driven by surface tension and Marangoni effects resulted in preferential oxygen redistribution from groove bases to edges, accompanied by phase transformations that converted surface Ti and Ti_2_O_3_ into stable TiO_2_. Wettability analysis demonstrated transient superhydrophilicity (contact angles <10°) in most specimens within 1–7 days post-processing, except for bioinspired egg-trap structures where organic contaminant redeposition altered surface properties.

At an area energy density of 5 μJ/μm^2^, rough disordered surfaces exhibited residual tensile stresses and trace TiN phases, while biological evaluations revealed significant roughness-dependent cellular responses. Surfaces with a roughness of 10.96 μm showed markedly reduced cell viability and inhibited proliferation despite their superhydrophilic nature, contrasting with optimal cellular adhesion and growth observed on surfaces with Sa = 5 μm. Topographical guidance effects were evident through preferential cell alignment along grooves with 2.91 μm edge radii compared to 7.75 μm counterparts, though quantitative analysis indicated no statistically significant differences in BMSC adhesion or proliferation rates between edge radius groups (p > 0.05). These findings underscore the multifactorial nature of laser-textured surface optimization, where biological performance depends on balancing oxidation states, controlled roughness parameters (Sa ≈5 μm), and mitigation of processing-induced defects, while highlighting the critical need to prevent organic contamination during fabrication. The study provides fundamental insights for developing laser-processed titanium alloy implants that synergize enhanced osseointegration with minimized adverse cellular responses through precision surface engineering (Xu et al., 2014; Sadlik et al., 2025).

## 2 Methods

### 2.1 The proposal of research ideas

**FIGURE 1:**
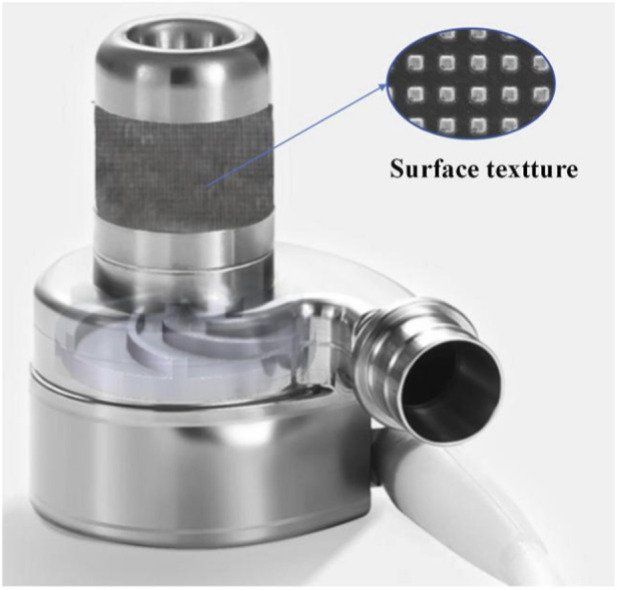
Schematic diagram of surface texture processing area on the surface of LVAD.

**FIGURE 2:**
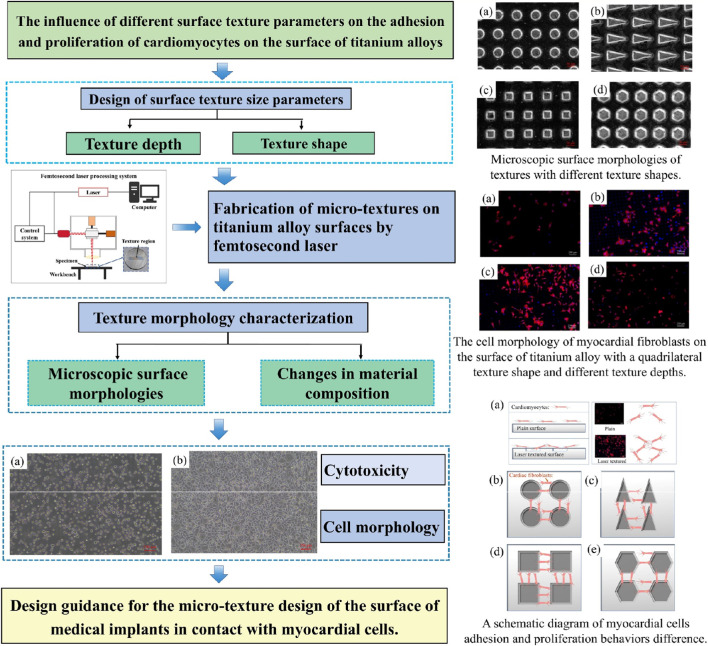
The technical roadmap of the study.

In this study, investigation would be conducted on the cell compatibility of the surface texture of the left ventricular assist device. Based on the surface polishing and laser surface texturing technology of titanium alloy, micron-sized texture patterns with uniform size and distribution were processed on the polished surface of titanium alloy. To explore the influence of different dimensional parameters, such as the texture shape and depth of micron-sized fabric patterns, on the blood compatibility of treated titanium alloy surfaces. Cell proliferation performance tests were conducted on the surface of specimens with different texture parameters using the mouse fibroblasts L-929. The fluorescent protein technique was used to characterize the distribution of cells and proteins under different surface texture parameters. Clarify the effects of different texture shapes and depths on the adhesion and proliferation of myocardial fibroblasts. Design the surface texture suitable for the application conditions of the left ventricular assist device and optimize the surface biocompatibility of the LVAD. As shown in Figure 1, the textured surface will be arranged at the contact areas between the LVAD entrance and the myocardium. The technical roadmap of the study is illustrated in Figure 2. This research aims to provide design guidance for the micro-texture design of the surface of medical implants in contact with myocardial cells.

### 2.2 Design of surface texture physical dimension parameters

**FIGURE 3:**
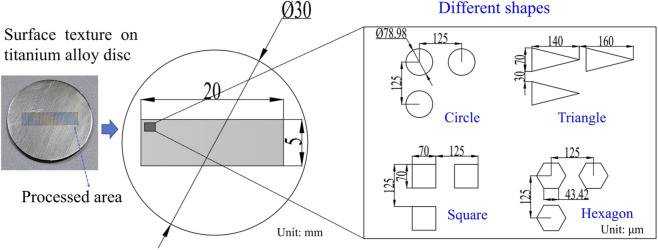
Schematic diagram of surface texture processing dimensions.

Figure 3 shows the dimensional parameters of the test specimen. The whole specimen is a titanium alloy disc with a diameter of 30 mm and a thickness of 2 mm. This diameter can be precisely placed in the six-well plate used for cell culture, which can reduce the impact of non-titanium alloy surfaces on cells. The rectangular part in the middle of the disc is the processing texture area, with a processing length of 20 mm and a width of 5 mm. The texture size ranges from the nanometer scale to the micrometer scale. This study mainly explores the effect of micrometer-scale texture on the adhesion and proliferation of RCF. The main research focuses on two parameter variables: texture depth and texture shape. The texture depths are selected at three levels: 10 μm, 20 μm, and 40 μm. The texture shapes are chosen with four types: circle, triangle, square, and hexagon. To control a single variable, the area ratios of the textures area of the four different shapes were all controlled at 31.4%.

### 2.3 Specimen preparation of titanium alloy surface texture

Ti-6Al-4V (TC4) titanium alloy has the characteristics of low density, corrosion resistance and good biocompatibility. It has been commonly used in various medical devices, including vascular stents, surgical instruments, and artificial heart components (Schweitzer et al., 2020; Potthoff et al., 2014; Lim et al., 2022; Huang et al., 2024). Therefore, the material used in the left ventricular assist device designed in this study is also TC4 titanium alloy, and medical-grade Ti-6Al-4V titanium alloy is used as the material for texturing the bottom surface. The chemical compositions of Ti-6Al-4V titanium alloy used in the study (wt%) are listed as follows: Al, 5.5–6.75; V, 3.5–4.5, C, ≤0.08; Fe, ≤0.3; N, ≤0.05; H, ≤0.015; O, ≤0.02; Ti, balance.

Surface texture using femtosecond laser processing (FLP) is an advanced micro-nano manufacturing technology. It utilizes the ultra-short pulses (1 femtosecond = 10^−15^ s) and ultra-high peak power characteristics of femtosecond lasers to precisely fabricate micron or even nanometer-level structures on material surfaces. Due to the extremely short pulse time of femtosecond lasers, energy is released in a concentrated manner within an extremely short period. After absorbing the energy, the material rapidly vaporizes or ionizes, hardly transferring heat to the surrounding area. Thus, thermal damage and melting phenomena are avoided, achieving “cold processing”. This technology can process regular micro-holes, grooves, protrusions, arrays and other structures on the surfaces of various materials such as metals, ceramics, glass and polymers. Meanwhile, due to its high precision, non-contact and wide range of applicable materials, it has become an important tool in modern precision manufacturing (Li et al., 2024; Wang et al., 2021; Brunette et al., 2001; Zhang et al., 2022; Ince and Özel, 2025).

**FIGURE 4:**
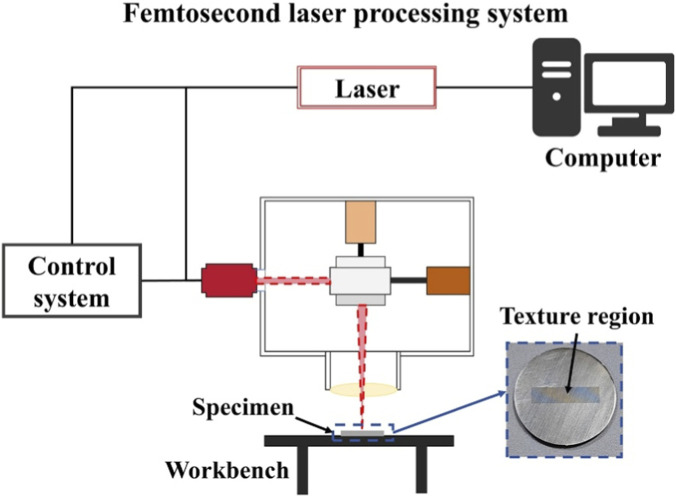
Schematic diagram of the surface texture processing of the TC4 titanium alloy specimen.

In this study, the GY-LC-03 FLP equipment of Changzhou Guangyi Laser Technology Co., Ltd. Was adopted for the surface texture processing of TC4 titanium alloy specimens. The processing parameters of the laser system used for surface texture processing are listed as follows: laser power is 5W, wave length is 1,030 nm, scanning speed is 200 mm/s, and frequency 500 kHz. After the laser processing was completed, the Ti-6Al-4V titanium alloy specimen was finely polished using 3200-mesh metallographic sandpaper. Figure 4 represents a complete schematic diagram of the apparatus for preparing surface texture specimens of TC4 titanium alloy specimen.

### 2.4 Microscopic observation of titanium alloy surface texture

To observe the processing quality of the surface texture of TC4 titanium alloy processed by FLP, the textured surfaces with different dimensional parameters were observed by using a tungsten filament scanning electron microscope (SEM, Quanta 250).

### 2.5 Cell proliferation performance test using mouse fibroblasts L-929

In this study, mouse fibroblasts L-929 were selected to conduct cell proliferation performance tests on different TC4 titanium alloys specimens. The effect of the test specimens on cell proliferation was evaluated by culturing mouse fibroblasts L-929 on the surface of TC4 titanium alloy with different texture parameters. Before conducting the cell proliferation test, the cells need to be cultured and passaged (Veiga et al., 2012; Phillips et al., 2015; Terakawa, 2018).

**FIGURE 5:**
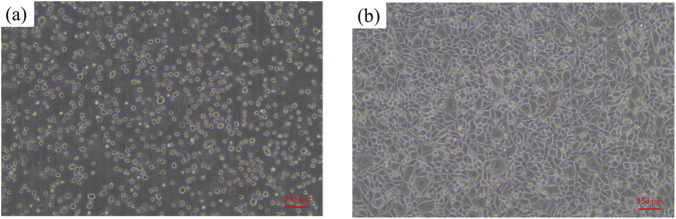
Mouse fibroblasts L-929 cell culture: **(a)** suspension cells; **(b)** cells in proliferation.

The cells used in this experiment were the mouse connective tissue L cell L-929 clone (L-929) cells of Taize (Guangzhou) Biotechnology Co., Ltd. and the resuscitation cells in the T25 bottle. The received cells were placed statically in an incubator at 37 °C and with 5% CO_2_ overnight. Unattached cells are shown in Figure 5a, and the cells were then subcultured. Figure 5b shows the L-929 cells in proliferation after subculture.

Completely aspirate the culture medium from the culture flask with a pipette, add 5 mL of PBS solution to rinse the cells, and then aspirate all the PBS solution. Add 2 mL of cell digestion solution (0.25% trypsin +0.53 mM EDTA), gently shake the culture flask to ensure that the trypsin solution fully covers the cell surface, and digest in the incubator for 3 min. After the cells become round and fall off, add 5 mL of the culture medium and use a pipette to draw the culture medium and pipette the cells adhering to the wall surface to make the cells fall off and disperse. Transfer the cell suspension into a sterile 15 mL centrifuge tube, centrifuge on a centrifuge, and centrifuge at 1,000 rpm for 5 min. The supernatant was aspirated with a pipette, and the cells were resuspended in 6 mL of medium. They were subcultured at a ratio of 1:2 and inoculated into new culture flasks (Choi and Schwarz, 2020).

### 2.6 Biocompatibility evaluation using MTT method

In this study, the 3-(4,5-dimethylthiazol-2-yl)-2,5-diphenyltetrazolium bromide (MTT) method was used to characterize the cell proliferation of cells cultured on different textured surfaces for biocompatibility evaluation. The principle of the MTT method is that the MTT dosage will be reduced by succinate dehydrogenase in the mitochondria of living cells to a blue-purple insoluble crystal, methazan. However, damaged cells lack the activity of this enzyme and are unable to carry out this reaction. During the MTT test, the cells were inoculated into Petri dishes. After appropriate treatment, MTT solution was added and the incubation continued for a period of time to enable the living cells to produce methazan crystals. Subsequently, a solvent such as dimethyl sulfoxide (DMSO) is added to dissolve the crystals, and the absorbance value is measured using an enzyme-linked immunosorbent assay (ELISA) reader (typically 570 nm or 490 nm). The proliferation rate and toxicity of cells can be evaluated by comparing the absorbance values of the treatment group and the control group (Poumellec et al., 2011).

The surface cell proliferation performance tests of TC4 titanium alloy specimens were divided into four experimental groups: the experimental group, the negative control group, the positive control group and the blank group. The experimental groups were TC4 titanium alloy with different shape parameters and surface textures, while the negative control group was TC4 titanium alloy with smooth surfaces. After disinfection with 75% ethanol and ultraviolet light, they were immersed in MEM medium. The positive control group was the culture Wells without titanium alloy specimens added. All specimens were cultured in a 37 °C incubator with 5% CO_2_ for 24 h and 48 h. The blank control group was the culture Wells where cells were placed in the medium but not cultured (Xin et al., 2022; Lin et al., 2018; Lu et al., 2008).

The entire operation is carried out within a laminar flow hood to ensure a sterile process. Cells that had grown to the logarithmic growth phase were digested with 0.25% trypsin (containing EDTA). After digestion, the cell suspension was centrifuged (1,000 rpm, 5 min), the supernatant was discarded, the cells were resuspended in MEM medium, and the cells were counted with a cell counter. The cells were diluted to 2 × 10^5^ cells/ml of cell suspension.

The cell suspension was inoculated into a 6-well plate at a rate of 1 mL per well to ensure that the cell suspension could completely cover the surface of the titanium alloy discs. And they were cultured in a cell culture incubator (37 °C, 5% CO_2_, >90% humidity), and the cell morphology of the positive group was observed by using a microscope. The positive group was the culture Wells without titanium alloy specimens, which was convenient for observing the growth of cells during the culture stage.

After culturing for 24 h and 48 h, respectively. The growth of the cells in the positive group without TC4 titanium alloy but with cells was observed under a microscope. Then the liquid was removed and the cells were rinsed with PBS solution. Mix the MTT and MEM media to form a 10% MTT solution. Add 1 mL of 10% MTT solution to each well in a 6-well plate and return the 6-well plate to the incubator. After culling for 4 h, the supernatant was removed, the cells were rinsed with PBS again, and then 1 mL of dimethyl sulfoxide solution was added. After thorough shaking, the absorbance value at a wavelength of 570 nm was determined on a microplate reader, the relative cell proliferation rate could be determined by Equation 1 (Gusenbauer et al., 2018).

$$RGN=\frac{D_{t}-D_{nc}}{D_{pc}-D_{nc}}\times 100\%$$ (1)

among them, *RGN* is the relative cell proliferation rate (%), and *D*_t_ is the absorbance of the well containing cells, culture medium, MTT solution and the soaking solution of the specimen to be tested; *D*_pc_ is the absorbance containing cells, culture medium and MTT solution. *D*_nc_ is the absorbance of the medium and MTT solution without cells.

### 2.7 Cell morphology and adhesion early behavior investigation using rat cardiac fibroblasts (RCF)

In the Section of 2.6, the MTT method was used to evaluate the effects of different texture parameters on the proliferation of mouse fibroblasts L-929. However, it can only analyse the impact of texture on cell proliferation at the macroscopic level. It is hard to further analyse its influence mechanism. Therefore, in order to further investigate the influence of different texturing parameters on the growth and distribution of rat cardiac fibroblasts (RCF), that is, to characterize the early adhesion behavior of cells on the textured titanium alloy surface and observe the distribution pattern of cells on the textured surface.

In order to observe the growth morphology of RCF on different textured surfaces, fluorescence microscopy or confocal microscopy is generally used for observation. Considering that the texture was processed on opaque titanium alloy discs in this study, a fluorescence microscope was chosen to observe the morphology of RCF (Willis et al., 2021; Piszczek et al., 2020).

The principle of fluorescence microscopy is that fluorescent dyes combine with specific cellular components, endowing them with specific fluorescence emission. Its fluorescence emits light of a specific color under light of a specific wavelength, ensuring the specificity of the fluorescence labeling and being able to well reflect the details of the stained cell components.

Cells cultured on the textured surface with different texture parameters were observed using the ECLIPSE Ni Series fully automatic upright fluorescence microscope (Nikon Precision Machinery (Shanghai) Co., Ltd.). In the aspect of Statistical Analysis, statistical analysis was performed on the observed results by using ImageJ software. There were 6 specimens (n = 6) in each group, and the results are expressed as mean ± SEM (Standard Error of the Mean). For multiple group comparisons, one-way ANOVA analysis was employed, followed by Tukey’s multiple comparisons test. The symbols “*” mean significant differences (p values less than 0.05); and the symbols “N. S.” indicate “Not Significant”.

This study mainly investigates the influence of surface texture depth (circular, triangular, square, hexagonal) and texture shape (10, 20, 40 μm) on the adhesion early behavior of cardiomyocytes. Therefore, 3 × 4 groups of specimens were designed for the experiment. Fluorescence staining was performed on the cultured RCF for observation. After taking the specimens out of the constant temperature incubator, the titanium alloy specimens were rinsed 2 to 3 times with PBS solution, each time for 3–5 min. Then, the cells were morphologically fixed with 4% paraformaldehyde solution for 10 min. After the cells were fixed, they were permeated with 0.5% deionized water (Triton X-100) for 30 min. Wash twice with PBS solution after taking it out. Since we mainly need to observe the distribution of cells and tissues, the nuclei and actin of the cells on the surface of the specimen are mainly stained. To stain F-actin fibres, FITC-gapenin was diluted with 1% artificial bovine serum albumin (BSA) PBS solution at a ratio of 1:100. The specimens were incubated together with the FITC-gapenin solution at 37 °C for 45 min. Then, use PBS solution to wash off the diluted gentamicin solution on the surface of the titanium alloy specimen. The cell nuclei were stained by incubating with 4′, 6-diamidino-2-phenylindole (DAPI) at 37 °C for 5 min. After cleaning off the DAPI staining solution on the surface of the specimen, it was observed under a fluorescence microscope.

## 3 Results and discussion

### 3.1 Observation results of surface texture morphology

**FIGURE 6:**
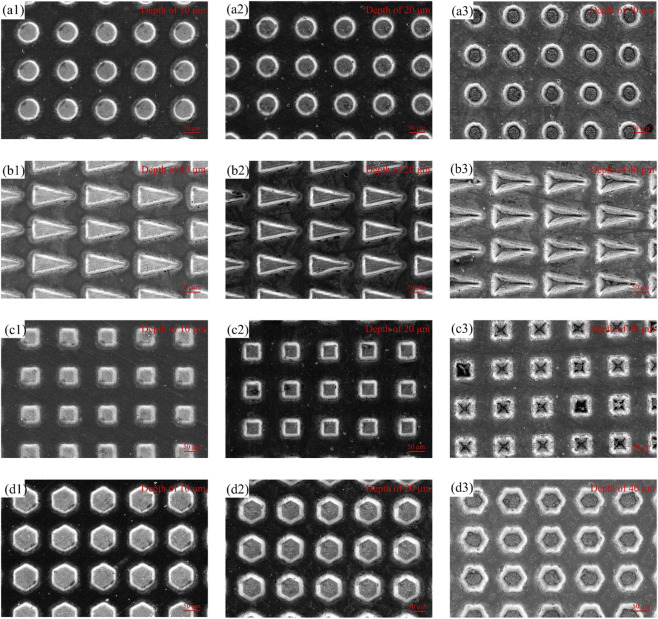
Microscopic surface morphologies of surface textures with different shapes: **(a1–a3)** circle with different depths; **(b1–b3)** triangle with different depths; **(c1–c3)** square with different depths; **(d1–d3)** hexagon with different depths. The texture depths of samples 1, 2 and 3 in each group are 10, 20 and 40 μm, respectively.

Figure 6 show the microscopic morphologies of the surfaces of textured TC4 titanium alloy with different dimensional parameters processed by FLP technology. Overall, FLP treatment has achieved reasonable surface texture processing quality in TC4 titanium alloy processing, with clear boundaries of individual textures and almost no impact on non-processed surface areas.

**FIGURE 7:**
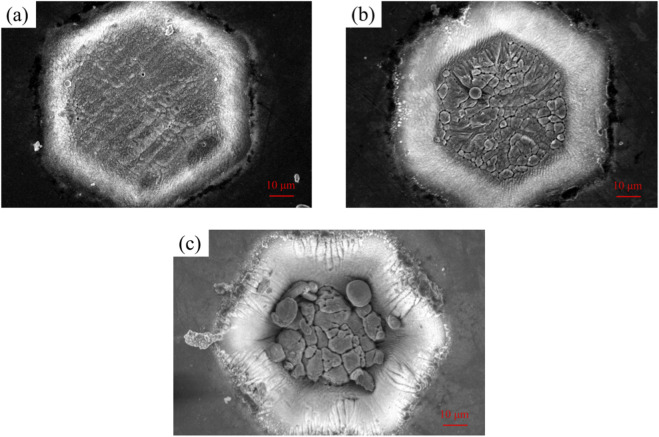
Magnified microscopic surface morphologies of a single texture with a hexagonal texture shape and different texture depths: **(a)** 10 μm; **(b)** 20 μm; **(c)** 40 μm.

Figure 7 shows the bottom magnification of the hexagonal texture formed at different processing depths. Observed under a high-power microscope, a raised microstructure was formed on the textured surface. In the regions with a lower texture depth, the restricted geometric space promotes the formation of regular convection cells, which explains why a periodic arrangement of microstructures is observed in this area. While in the regions with a higher texture depth value, the stronger inertial force leads to unstable and transient turbulence, it could be supposed that Marangoni convection and buoyancy convection are caused to compete with each other during the femtosecond laser processing (Wang et al., 2021; Lin et al., 2021; Gurevich, 2016), ultimately resulting in the disordered and mottled morphology observed in Figure 7. As the texture depth increases, the deepening of the texture depth will require a higher energy density, which in turn leads to an increase in the temperature of the processed surface. During the ablation process, the vaporization of the internal elements is not uniform. The molten liquid generated during the laser processing, when mixed with the ablation plume, will present a sputtering state. After solidification, it will present a new and uniform texture.

By comparing the bottom textures processed by FLPs at different depths, the deeper textures will keep the processing area in a molten state. Due to the effect of Marangoni convection, the microstructure units would be further roughened. This phenomenon indicates that the texture depth has a significant influence on the formation and characteristics of microstructures (Cunha et al., 2016).

### 3.2 Characterization of material composition changes

**FIGURE 8:**
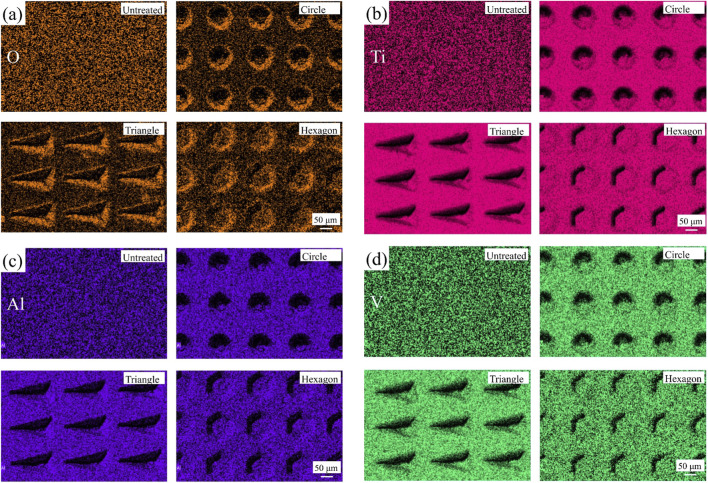
Distribution of four elements on the specimen surface with different textures: **(a)** oxygen; **(b)** titanium; **(c)** aluminum; **(d)** vanadium.

The distribution of oxygen, titanium, aluminum and vanadium elements on the surface of TC4 titanium alloy specimens with different texture shapes is shown in Figure 8, respectively. According to the distribution of oxygen elements, the oxygen elements on the smooth surface of titanium alloy are evenly distributed, while the oxygen elements on the surface of titanium alloy processed and textured by FLP show the effect of distribution almost along with the texture shape. Oxidation reactions occurred on the surface of TC4 titanium alloy during FLP, and a large amount of metal oxides were formed on the surface of TC4 titanium alloy processed by FLP. According to the literature (Cunha, 2015; Shaikh et al., 2019), the unsaturated metals and oxygen atoms on the surface of metal oxides constitute the Lewis acid-base active sites. These sites can rapidly adsorb water molecules in the air, significantly enhancing the hydrophilicity of the material’s surface. This mechanism enables metal oxides to exhibit excellent performance in improving surface hydrophilicity. Therefore, in addition to the surface structure morphology, using a FLP to process the texture on the surface of TC4 titanium alloy will make the surface have better hydrophilicity, and cells are more likely to adhere on the surface with better hydrophilicity (Li et al., 2024; Raimbault et al., 2016). In addition, it could be noted that outside the processed area on the specimen spacing surface, the distribution of titanium, aluminum and vanadium elements was not significantly affected.

### 3.3 Biocompatibility evaluation results

The biocompatibility of different textures was evaluated by conducting cell viability tests on mouse fibroblasts L-929. Cells were directly cultured on the specimen surface for a relative short period of time (24 h) or a longer period of time (48 h), and then the cell survival rate was detected using the MTT method. The higher the absorbance value is, the higher the cell survival rate will be. The lower the absorbance value is, the lower the cell survival rate will be (Lisjak et al., 2023).

**FIGURE 9:**
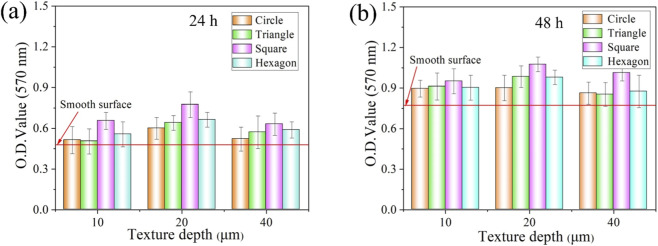
Relative cell proliferation rates of TC4 titanium alloy surface with different texture parameters after different culture time: **(a)** 24-h; **(b)** 48-h.

The cell proliferation rate of mouse fibroblasts L-929 cells cultured with different texture parameters are shown in Figure 9. Compared with the smooth surface (untreated) specimen group, the cell proliferation rate on the textured specimen surface has been significantly increased. According to the results of the cell proliferation test, it can be known that the surface texture is conducive to the proliferation of cells on the surface of TC4 titanium alloy. Combined with the analysis of the surface microscopic morphology after laser processing, a certain degree of roughness can promote aggregation in this area and provide points for cell adhesion.

Figure 9a shows the 24-h relative cell proliferation rates of titanium alloy surfaces with different texture parameters. Specifically, the proliferation of mouse fibroblasts L-929 cells on the surface of each specimen after 24 h of culture, and the optical density (O.D.) value of fibroblasts cultured from specimens with each surface texture at a wavelength of 570 nm. The values are all higher than the O.D. values of the smooth surface. This indicates that the textured surface of titanium alloy is more conducive to the proliferation of mouse fibroblasts L-929 cells compared with the untreated smooth surface of titanium alloy. In the design of surface texture, the influence of the depth and shape of the texture on cell adhesion and proliferation is mainly considered. Among the three texture depth parameters of 10 μm, 20 μm and 40 μm, when the texture depth is 20 μm, The O.D. value was significantly higher than that of the other two groups, which indicates that a texture depth of 20 μm is more conducive to cell proliferation. Meanwhile, among the four texture shapes, the O.D. value of the square texture group was significantly higher than that of the other three groups. The square texture shows a more obvious promoting effect on cell proliferation, while the hexagonal texture has a promoting effect on cell proliferation second only to the square texture. The promotion of cell proliferation by circular texture and triangular texture is relatively the same.

Figure 9b shows the relative cell proliferation rates of titanium alloy surfaces with different texture parameters for 48 h. It is found that after the mouse fibroblasts L-929 cells were continuously cultured for 48 h, the proliferation of surface textured cells with different parameters in each group was almost the same as that after 24 h of culture. However, the cells after 48 h of culture proliferated more than those after 24 h of culture.

These results are consistent with the common trend observed in other studies, that is, the surface micro-textures generated by laser texture processing can widely and uniformly expand the cell adhesion pattern, ultimately enhancing cell adhesion and improving biocompatibility. Wang et al. (2021) observed that, compared with the rough surfaces produced by traditional methods, osteoblasts on the laser-processed surface texture of Ti-6Al-4V specimens significantly improved adhesion and growth. Compared with the untreated and mechanically rough-treated specimens, the specimens treated with CO_2_ laser showed significant improvements in the proliferation and adhesion of rat fibroblasts. FLP treatment of micro/nano structures induced on the surface of Ti-6Al-4V plays a significant role in determining cell adhesion, proliferation and differentiation. High surface roughness can have a negative impact on cell adhesion and proliferation. On the other hand, overly rough surfaces may endanger the integrity of the cell membrane, leading to irreversible rupture of the cell membrane.

### 3.4 Cell adhesion early behavior analysis by morphology characterization and comparison

#### 3.4.1 Square texture shape with different texture depths

**FIGURE 10:**
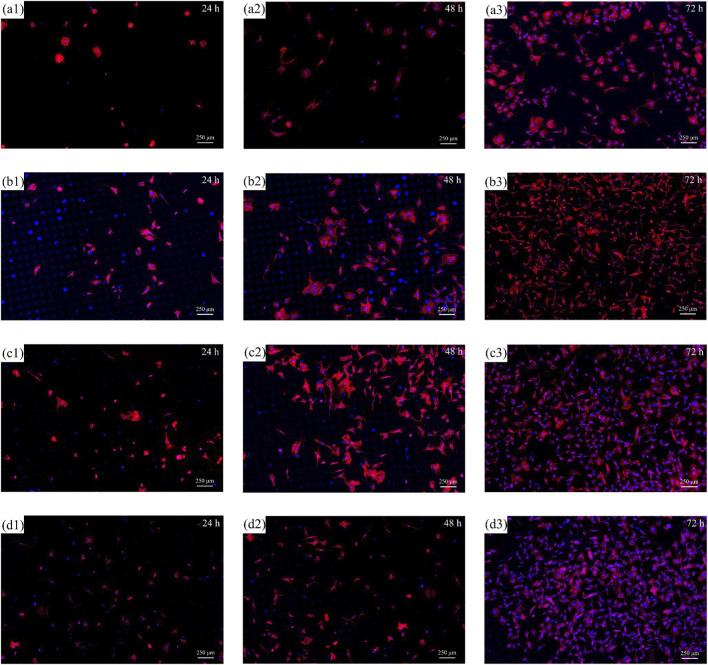
The cell morphology of RCF on the titanium alloy surface with a square texture shape and different texture depths: **(a1–a3)** untextured with different days; **(b1–b3)** depth of 10 μm with different days; **(c1–c3)** depth of 20 μm with different days; **(d1–d3)** depth of 40 μm with different days.

It can be known from the results of cell proliferation experiments with different texture parameters that the square texture has a relatively significant promoting effect on the proliferation of mouse fibroblasts L-929 on TC4 titanium alloy surface. Therefore, the square was determined as the shape of the texture, and the effects of different texture depths (untreated of 0 μm, 10 μm, 20 μm, 40 μm) on the proliferation behaviors of RCF were discussed. Figure 10 show the RCF distribution of textures at different depths when cultured for 1 day, 2 days, and 3 days.

The distribution of RCF cells on the texture surface at different depths was observed, which was similar to the mouse fibroblasts L-929 cell proliferation situation. The textured surface has a higher number of RCF cells distributed compared to the smooth surface, and this situation was more significant in the previous 2 days. Since RCF cells began to adapt to the culture environment on the first day of culture, conducting initial adhesion and extension, the proliferation activity has not been significantly enhanced yet. The cellular activities of textures at different depths were all relatively weak. However, since the textured surface can provide more adhesion points for the adhesion of RCF, the smooth surface had less cell distribution compared to the textured surface after the first day of culture. The study also found that the number and morphology of cells distributed on the texture surfaces with texture depths of 10 μm, 20 μm, and 40 μm were relatively similar.

On the second day of RCF culture, the cells gradually stretched into a spindle-shaped or flat shape, with oval-shaped nuclei and clear nucleoli. The cell density increases, and contact begins to occur between cells, but a dense reticular structure has not yet formed. Cell proliferation activities began to intensify, and the number of cells gradually increased. The cell distribution on the smooth surface is still significantly less than that on the textured surface. After comparing the distribution of RCFs with different texture depths, it was found that when the texture depth was 10 μm, more contact points were provided for the adhesion of cells. However, due to the relatively shallow texture depth, the growth direction of cells is not defined by the texture. Therefore, the cell morphology distributed on it is similar to that on a smooth surface, while the deeper texture may affect the cell growth morphology, making the cell growth have a certain directionality. When the texture depth is 20 μm, its cell proliferation is better than that of the other two texture depths.

When RCF cells are cultured to the third day, the RCF cell morphology becomes more stable, presenting as spindle-shaped or flat, and a reticular structure is formed between the cells. The cell density further increases, and the contact between cells becomes closer. Cell proliferation activities were significantly enhanced, and the number of cells increased significantly. In some areas, cell fusion phenomena may begin to occur. Still, the number of cells proliferating on the textured surface was higher than that on the smooth surface. However, when comparing the surfaces of specimens with different texture depths, on the surfaces of TC4 titanium alloy with texture depths of 20 μm and 40 μm, there are more cell distributions, and fusion growth occurs in some areas.

**FIGURE 11:**
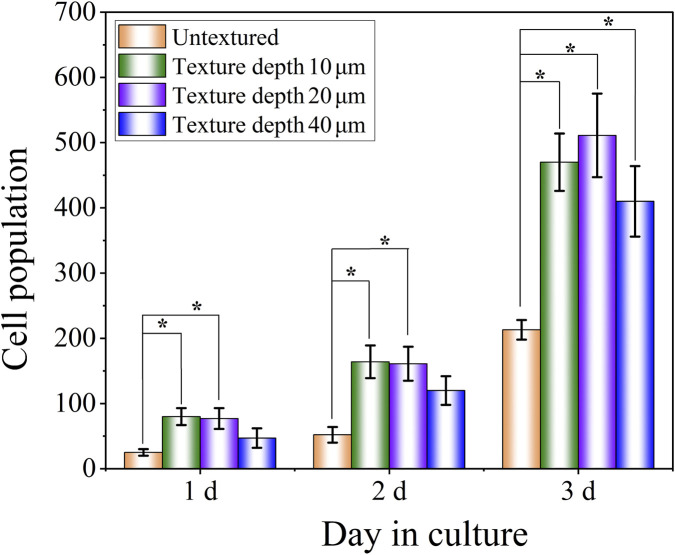
Statistical results of the number of RCF cells of TC4 titanium alloy square texture shape with different texture depths.

The number of RCF cells cultured on the surface of TC4 titanium alloy with different texture parameters for 1, 2 and 3 days was statistically analysed using the ImageJ software. To ensure the reliability of the counting results, the number of cells within a 5 mm^2^ area in the statistical field of view was selected and counted three times at different positions of the same specimen. It can be known from the results in Figure 11 that the number of RCF cells increased most significantly on the third day. Corresponding to the observation results of cardiomyocyte morphology, the number of cells in specimens with different texture depths increased significantly compared with that in untextured specimens (*p* values less than 0.05). The number of cells with a texture depth of 40 μm was lower than that of the other two groups because a deeper texture depth would lead to an increase in texture roughness and thereby affect cell proliferation behavior.

It is found that overly deep textures (such as depth of 40 μm) restrict the physical contact between cells and the bottom of the grooves, significantly reducing the effective adhesion area. At the same time, RCF cells overextend to cross the deep grooves, which is possible cause imbalance in the cell skeleton tension (Wang et al., 2021). Moreover, the deep groove structure may form a local quality transmission barrier. In summary, when the depth exceeds the critical value, the role of the surface texture changes from “guidance” to “restriction”, thereby inhibiting RCF cell activity.

#### 3.4.2 Texture depth of 20 μm with different texture shapes

**FIGURE 12:**
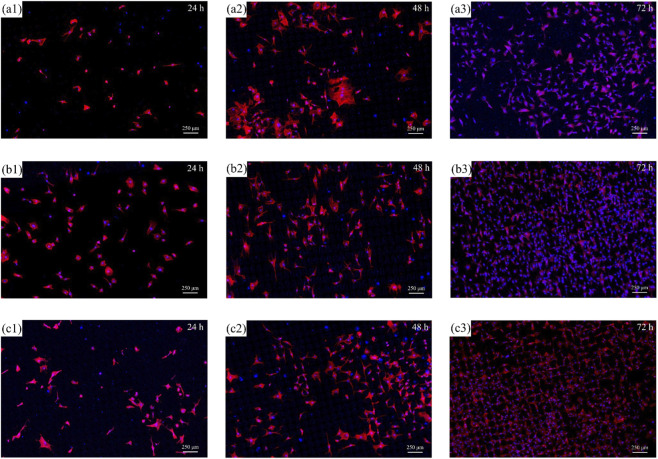
Cell morphology of RCF on the surface of titanium alloy with a texture depth of 20 μm and different texture shapes: **(a1–a3)** circle with 24 h, 48 h, and 72 h; **(b1–b3)** triangle with 24 h, 48 h, and 72 h; **(c1–c3)** hexagon with 24 h, 48 h, and 72 h.

To further clarify the influence of texture shape on cell adhesion behavior, single-variable experiment and analysis was also conducted. It can be known from the cell proliferation test results of different texture parameters and the cell morphology observation results of different texture depths that the texture with a texture depth of 20 μm has a relatively significant promoting effect on the proliferation of fibroblasts in TC4 titanium alloy. Therefore, 20 μm was determined as the texture depth value for discussing the effect of different texture shapes on the adhesion behavior of RCF, and the influence of different texture shapes (circle, triangular, square, hexagonal) on the proliferation behavior of RCF was discussed. Figure 12 shows the cell distribution of textures of different shapes when cultured for 1 day, 2 days, and 3 days.

The proliferation of RCF on the surfaces of TC4 titanium alloy with different texture shapes and textures with different texture depths was generally similar, all reaching the fastest proliferation rate on the third day. However, the textures of different shapes show that the texture has a significant influence on the direction of cell proliferation.

The multi-level morphology formed by the FLP technology significantly increases the contact area between cells and TC4 titanium alloy. Meanwhile, the randomness of this form also leads to the random distribution of cell orientations. On a textured surface, the orientation of cells is significantly influenced by the texture shape. Similar results have been also reported by Wang et al. (2021), they found that the anisotropic surface characteristics prompt the focal contacts to align along the direction of high surface energy. The total potential energy consumed by cell activities is minimized along the surface texture direction, which enables the cells to exhibit good orientation. In this case, the aspect ratio of the cells is significantly increased, and the orientation of microfilaments along the groove direction is enhanced, thereby promoting the rearrangement of the cytoskeleton. The cytoskeleton undergoes rearrangement on surfaces with different surface energies, resulting in complex dynamic changes in the tension generated by the cells. The adhesion force between cells and the textured surface will be higher. However, the disorder of surface morphology and height differences may also lead to the disordered arrangement of microfilaments, increasing the potential energy consumed by cells and thereby weakening cell activity. Therefore, different cell types have different fitness values for the surface energy and roughness of implants. In addition, the interfacial tension between cells and materials also directly affects the intracellular tension, cell membrane tension, and the morphology of the cytoskeleton.

**FIGURE 13:**
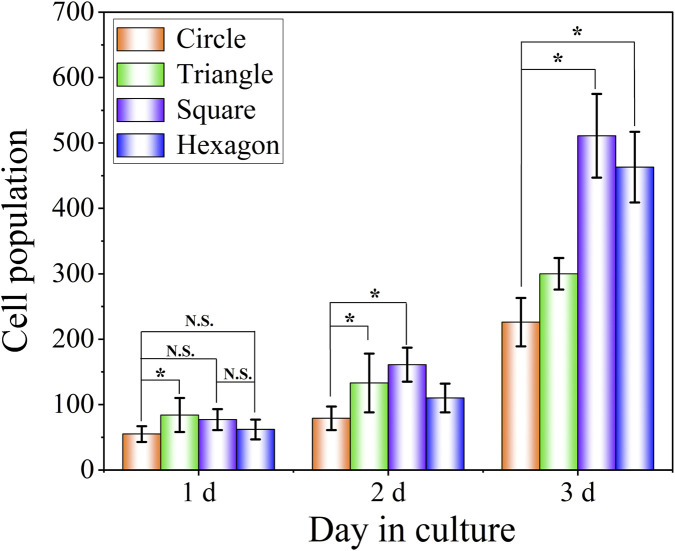
Statistics of the number of surface cells of TC4 titanium alloy with different texture shapes.

The number of cell proliferation after culturing RCF on the textured surface of different textured shapes for 1, 2 and 3 days was statistically analysed. To ensure the reliability of the counting results, the number of cells within a 5 mm^2^ area in the statistical field of view was selected and counted three times at different positions of the same specimen. According to the statistical results in Figure 13, it is not significant between the different texture shapes on the 1-day culture. With the culture time increasing, it can be known that the number of cells on the texture surfaces of the square texture and the hexagonal texture is significantly higher than that of the other two groups. Due to the certain size effect limitations of cell growth, the spacing between individual textures of the designed square texture and hexagonal texture could better meet the adhesion and growth of RCF. However, there are more obvious differences of the spacing of the circle and triangular textures, and the proportion of the appropriate spacing that can promote cell proliferation is at relatively small level. Therefore, the square texture exhibits the best promoting effect on cell proliferation, followed by the hexagonal texture.

**FIGURE 14:**
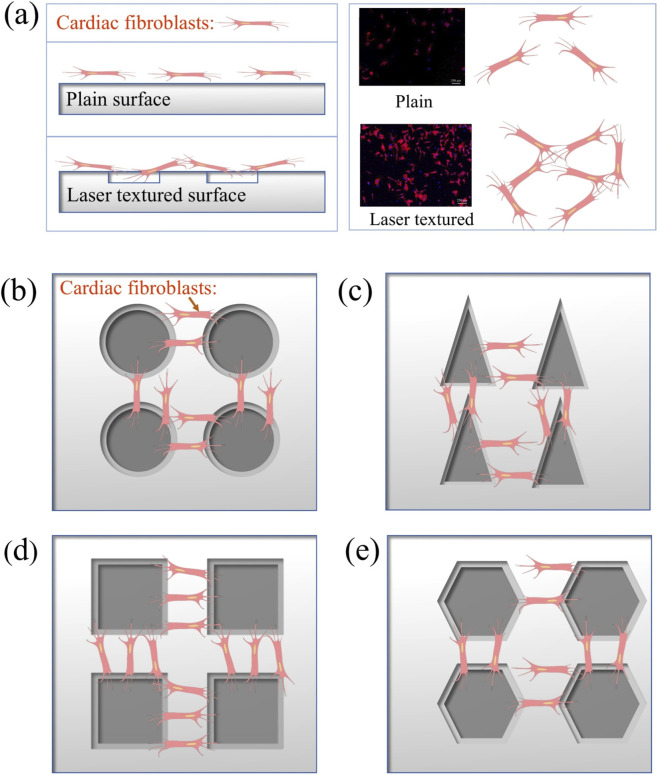
A schematic diagram of RCF cells adhesion and proliferation behaviors difference on the untreated surface and four types of shape surface texture: **(a)** the comparison of untreated surface and textured surface, **(b)** circle shape, **(c)** triangle shape, **(d)** square shape, **(e)** hexagon shape.

Based on the experimental results and the elaboration of related academic journal papers (Piszczek et al., 2020; Maalouf et al., 2022), different surface texture shapes have different adhesion and proliferation abilities of RCF on the surface of TC4 titanium alloy. Figure 14 illustrates a schematic diagram of RCF cells adhesion and proliferation behaviors difference on the untreated surface and four types of shape surface texture. Compared to the plain surface, the cell adhesion alignment, agglomeration and proliferation behaviors were promoted along with the surface texture due to its higher surface areas. The distance between the two square textures is uniform, while the triangular one has a larger range of variation. Therefore, the square texture shows the better results in biocompatibility and RCF cell adhesion than the other three texture shapes.

## 4 Conclusion

The conclusions in the study could be drawn as follows:

1. It can be known from the experimental results that the textured surface processed by laser will significantly affect the proliferation of cells on the specimen surface and change the growth and diffusion pattern of rat cardiac fibroblasts. From the comparison of macroscopic cell proliferation in the cell proliferation test, it can be seen that an appropriate texture depth can promote cell proliferation. When the texture depth is 20 μm, its ability to promote cell growth is significantly better than that of textures with texture depths of 10 μm and 40 μm. Meanwhile, different texture shapes also have a significant impact on the cell proliferation status. According to the experimental results, the square texture has the best promoting effect on cell proliferation, followed by the hexagonal texture.
2. The proliferation of cells on the textured surface is affected by the cell growth size effect. The distance between individual textures has a significant impact on cell early adhesion and proliferation. Through the morphological observation and evaluation experiment of rat cardiac fibroblasts, it can be observed that the textured surface can attract cell adhesion through regular, tiny rough surfaces in the early stage of cell culture behavior, thereby affecting the proliferation of cells on the textured surface in the later stage. Meanwhile, different texture parameters have a significant influence on the growth morphology of cells. A deeper texture (with a texture depth of 40 μm) will cause cells to proliferate along the single texture edge. These phenomena are more obvious in the later stage of cell culture.

## 5 Future perspectives

This study investigated the influence of surface texture on titanium alloy on the adhesion and proliferation of cardiomyocytes. However, this study only explored the effects of texture depth and texture shape, while the dimensional parameters of texture also include area ratio and arrangement mode, which need to be further studied. Meanwhile, in order to investigate the influence of surface texture on titanium alloy on the adhesion and proliferation of cardiomyocytes, this study mainly used cytotoxicity and fluorescent protein technology for analysis and characterization. In the further research, more advanced characterization techniques can be used to deeply analyze the adhesion influence mechanism. For example, involvement of key signaling pathways such as integrin focal adhesion kinase (FAK) pathway and Rho family GTPase pathway to explain the cell behavior.

## Data Availability

The raw data supporting the conclusions of this article will be made available by the authors, without undue reservation.
